# Supplementary material for: Exploring the Structure–Performance Relationship of Sulfonated Polysulfone Proton Exchange Membrane by a Combined Computational and Experimental Approach
Source: Polymers (Basel). 2021 Mar 20;13(6):959. doi: 10.3390/polym13060959 (PMC8003876; doi:10.3390/polym13060959)
Supplement: Supplementary file 1 [file polymers-13-00959-s001.pdf]

Supporting information

Exploring the structure-performance relationship of sulfonated polysulfone proton exchange membrane by a combined computational and experimental approach.

Cataldo Simari[a,*], Mario Prejanò[a,*], Ernestino Lufrano[a], Emilia Sicilia [a] and Isabella Nicotera[a]

a) Department of Chemistry and Chemical Technologies, University of Calabria, Via P. Bucci 14/D, 87036 Rende (CS), Italy

* cataldo.simari@unical.it; .mario.prejano@unical.it.

Corresponding authors

Figure S1 illustrates the surface and cross-sectional SEM images of the sPSU membrane. The film exhibits a dense, compact and homogeneous structure. Furthermore, the sPSU membrane presents a smooth and uniform morphology without any cracks and/or defects. This clearly indicates fine quality.

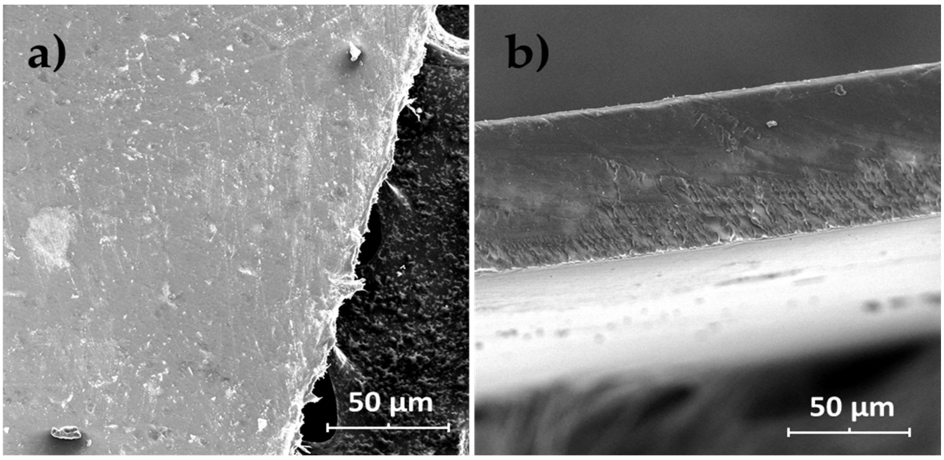

Figure S1. (a) Surface and (b) cross-sectional SEM images of the SPSU membrane.

In Figure S2 are presented the structures extrapolated from preliminary molecular dynamics on polymer containing 9 chains (90 monomers, 72 SO_3 groups) at 80 °C, 100 °C and 120 °C. This preliminary investigation was necessary since conformational information about the membrane were not available. 100 ns of molecular dynamics have been performed on these systems and, after analysis of the trajectories, the sampled conformations have been successively adopted as input in the creation of bigger models containing 270 residues (270 monomers, 216 groups).

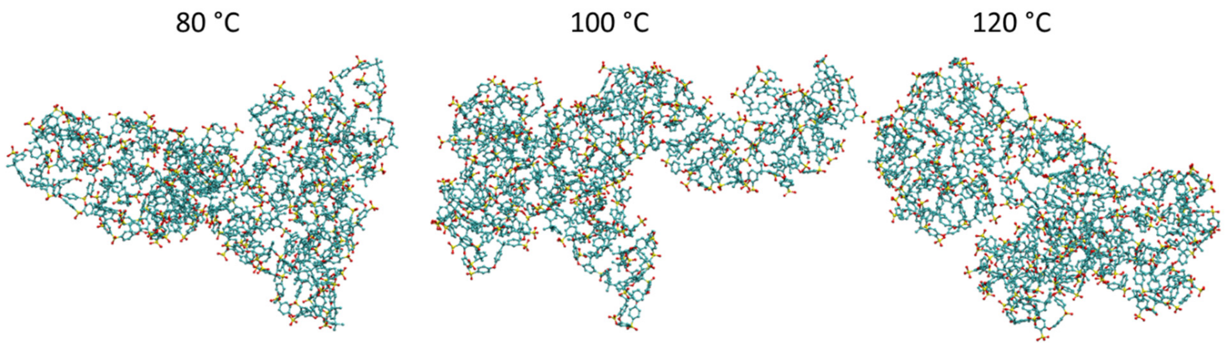

Figure S2. Most populated sampled structures extrapolated along the MD simulation of the model containing 9 polymer chains adopted as initial inputs in the buildup of the model containing 270 residues.

Figure S3 shows the initial guess geometry adopted in further MD simulations. The structure was obtained packing 270 monomers and water molecules in a fictious box.

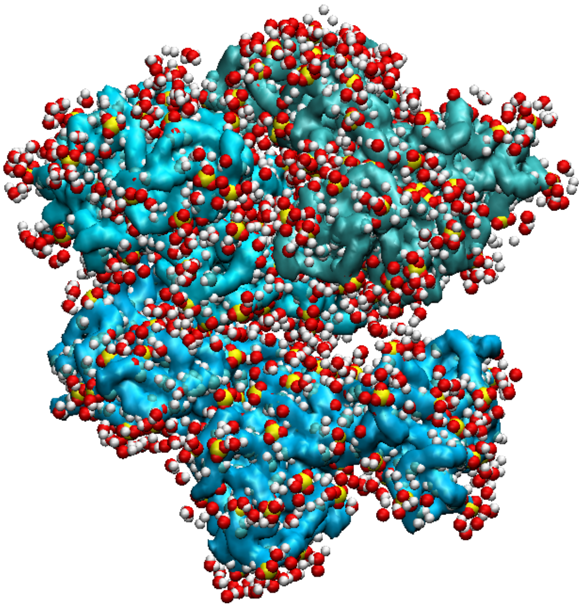

Figure S3. Initial structure adopted in the molecular dynamics simulations. For clarity, solely water molecules within 5 Å from the $-SO_3^-$ are represented.

Figure S4 presents the evolution of Root Mean Square Deviation, along the molecular dynamics, of C atoms composing the polymer. It can be note as, after an initial phase, the dynamics can be considered at equilibrium. The values highlight that the rearrangement occurring during the simulation and discussed in the manuscript was obtained after relevant shift, respect to the initial positions, of each residue composing the monomer.

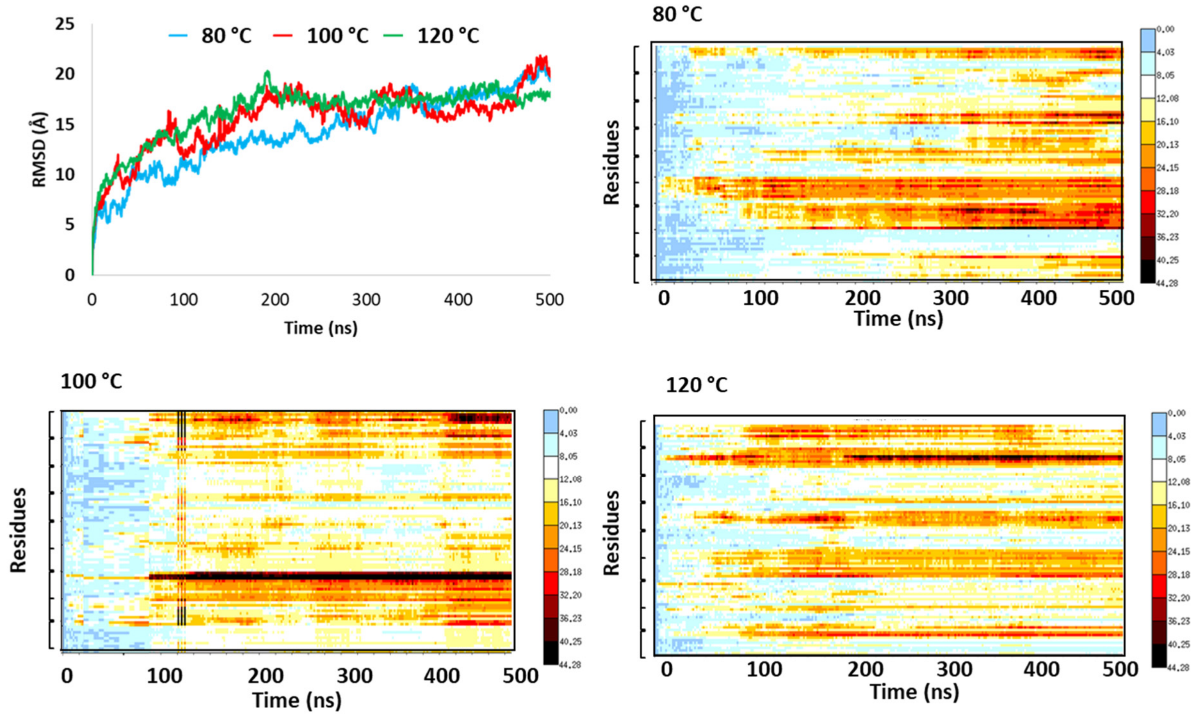

Figure S4. Root mean square deviation calculated for the all the C atoms (top-left) and for each monomer at the three different temperatures.

The RDFs presented in Figure S5 emphasize the trend owned by the aromatic rings, composing the backbone of the polymer, to pair with other benzene-containing moieties. In particular, can be note as ring R1 and R4 establisher pi-pi pairing interaction with two and three aromatic rings, respectively, along the dynamics, further supporting the phase hydrophobic-hydrophilic separation characterizing this polymer.

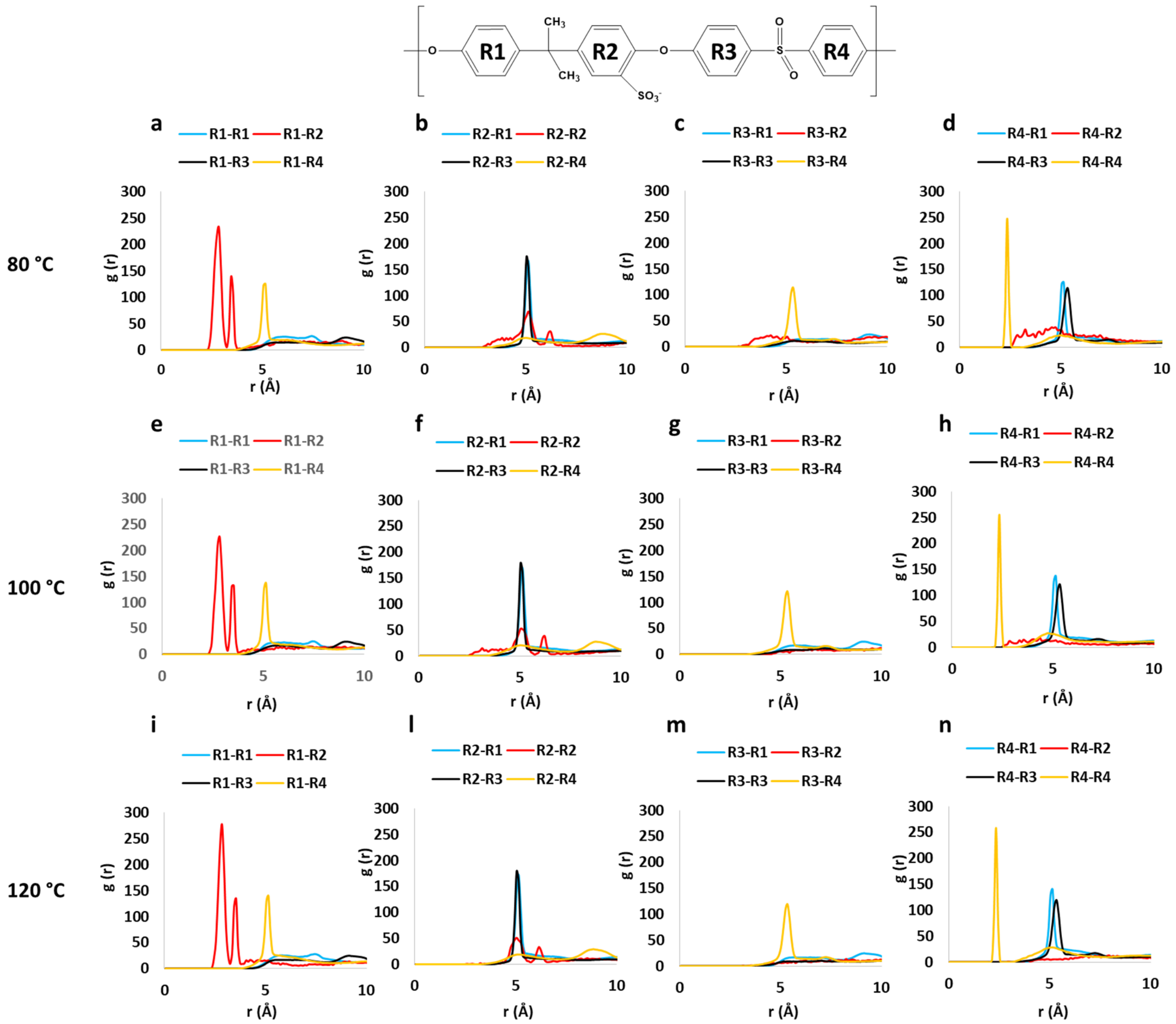

Figure S5. Pair correlation functions calculated along the MD simulations of model containing 9 chains between the centroids of the aromatic rings composing the sPSF monomer.

The model isolated from the MD simulation at 80 °C, and adopted for further DFT investigation, contains two sPSU monomers, with one SO_3 group respectively and a cluster of 10 interconnected water molecules. The atoms labeled with "*" were kept frozen during the optimization, in order to prevent artificial movements of atoms composing the backbone of the polymer. The remaining atoms were left free to optimize.

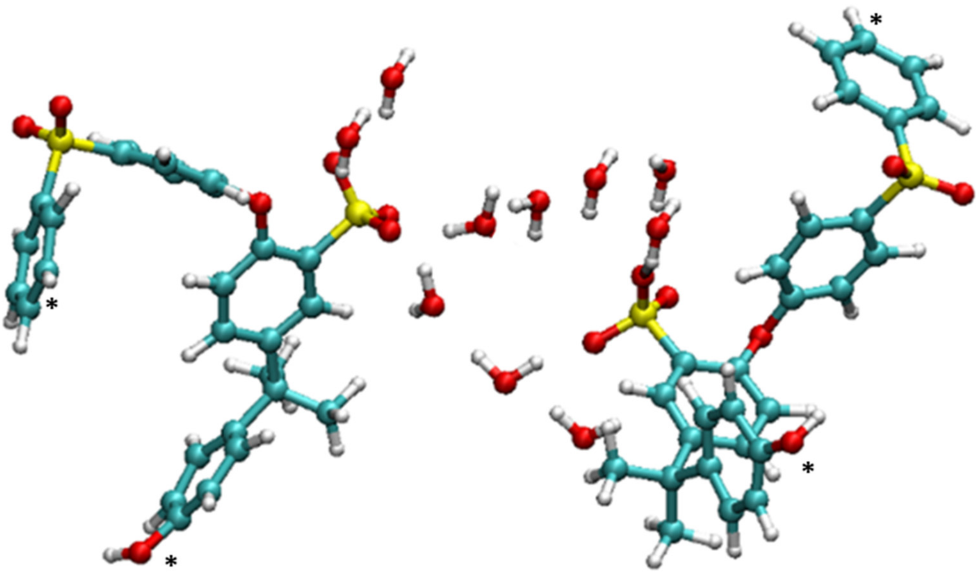

Figure S6. Model adopted for DFT optimization.

Figure S7 (a) shows the temperature evolution (from 20 up to 130 °C) of the ^{1}H spectra acquired on the sPSU membrane, for two representative water content, i.e. at saturation and 10 wt%. Proton spectra, referenced against pure water set at 0 ppm, were acquired with the same number of scans to compare their intensities. Clearly, both proton signals are very wide, implying a strong nanoconfinement of water molecules within the hydrophilic pores of sPSU. De facto, the corresponding linewidths (Fig. S7 c) range between 700 and 1300 Hz depending on the initial water content, typically reflecting a solid-like configuration. Additionally, the signals are very asymmetric being the convolution of "different" water signals (free and bound to the SO_3^- groups of sPSU) in fast rate of proton exchange. [1] Due to water evaporation from the membrane, the peak intensity progressively decreases during heating. In this regard, Figure S7d shows the temperature variation of the peak area, normalized to the initial water uptakes. For the 38-20 wt% of uptakes, the larger the decrement in the signal intensity can be seen above 60-80 °C, suggesting a greater contribution of free water (more mobile) to these NMR signals. Contrariwise, the signal loss is almost negligible for sPSU equilibrated at 10 wt% of water, indicating most of the water molecules are into a "bound state".

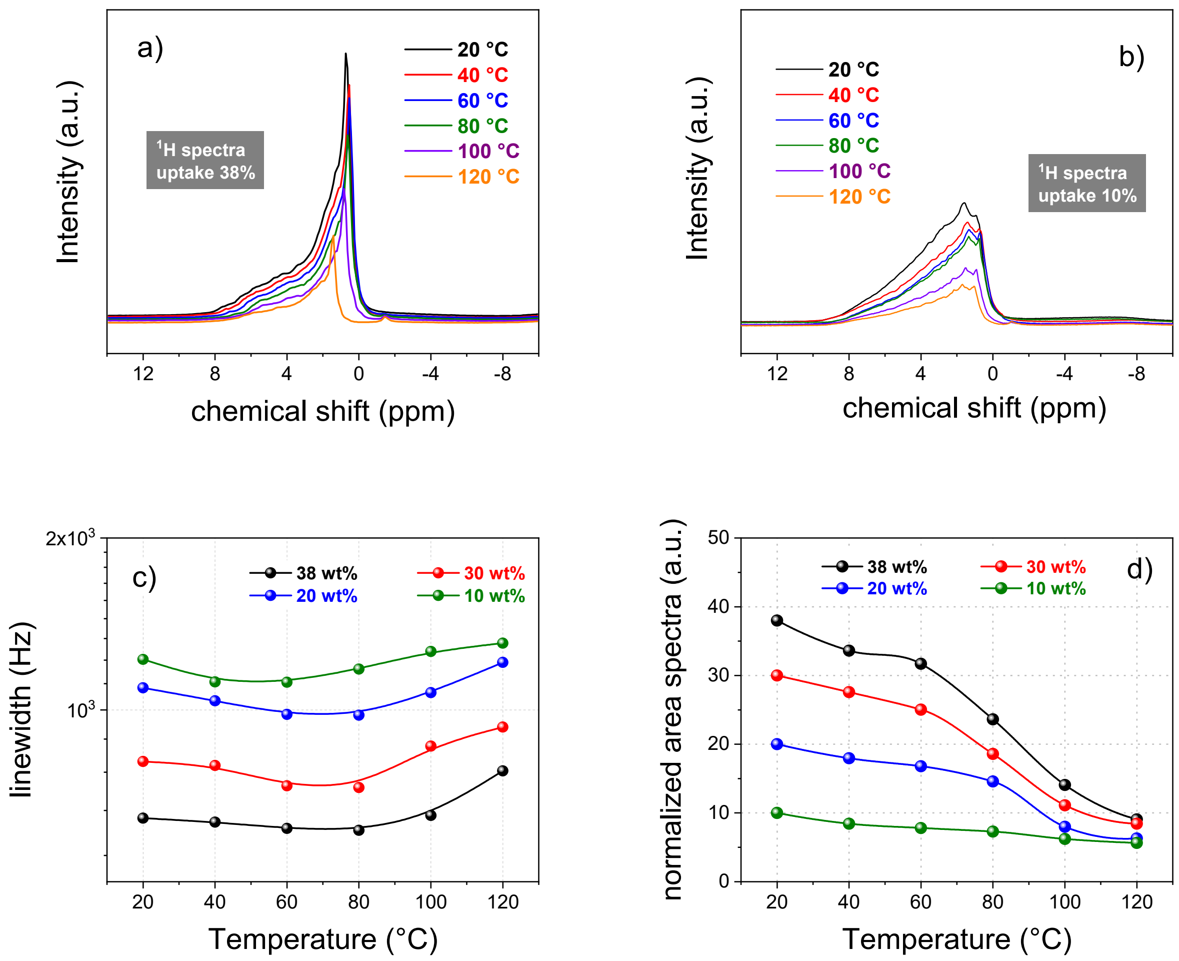

Figure S7. Temperature evolution, in the range 20-120 °C, of ^{1}H NMR spectra of the water confined in sPSU at (a) saturation and (b) 10 wt% of water. (c) Linewidth vs. temperature and (d) peak areas normalized to the initial water uptakes vs. temperature for the different water content.

In Table S1 are reported the population (%) of clustered structure of each MD simulation. Among the thousands of structures encountered during the simulation, was possible to isolate most populated geometry representing more than the 50% of molecular dynamics (the cluster 1), adopting geometrical hierarchical clustering procedure. The results indicated the good conformational homogeneity reached by the polymer in the course of the simulation.

Table S1. Frequency (%) of structures sampled during the hierarchical clustering procedure on a total number of 250000 frames extrapolated along the molecular dynamics simulations of model containing 270 residues.

CLUSTER	80 °C	100 °C	120 °C

**1**	58.8	50.4	56.7
**2**	10.1	15.2	8.0
**3**	6.6	11.4	7.3
**4**	6.5	5.9	6.6
**5**	5.6	4.4	5.5
**6**	3.4	3.7	4.3
**7**	3.3	3.6	3.3
**8**	2.3	3.3	3.2
**9**	2.0	1.5	2.6
**10**	1.4	0.7	2.4

Model setup

The setup of sulfonated polysulfone model presented in the manuscript proceeded through preliminary investigations on structural behaviors of smaller polymer model, constituted by 10 monomers, reported in Scheme S1, as follows:

- One terminal sulfonated molecule representing the head of the chain (HEA), presenting SO_3 group;
- Two molecules without SO_3 group (NS3), retained to simulate the sulfonation level equal to 80%;
- Six molecules presenting SO_3 group (MOL);
- One terminal sulfonated molecule representing the tail of the chain (TAI), presenting SO_3 group.

This initial model contained 8 SO_3 groups. The parameters of each residue have been extrapolated according to standard procedure mentioned in the main manuscript (see Molecular models section).

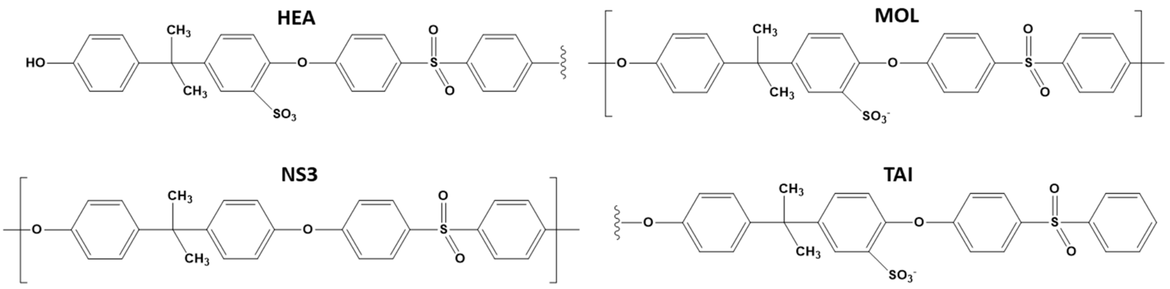

Scheme S1. Schematic representation of monomers adopted in the built up of the model.

The entire system was initially solvated, minimized, as depicted in Figure S8, and later has undergone to 50 ns of molecular dynamics in NPT conditions, at the fixed temperature of 20 °C, as analogously reported in the manuscript .

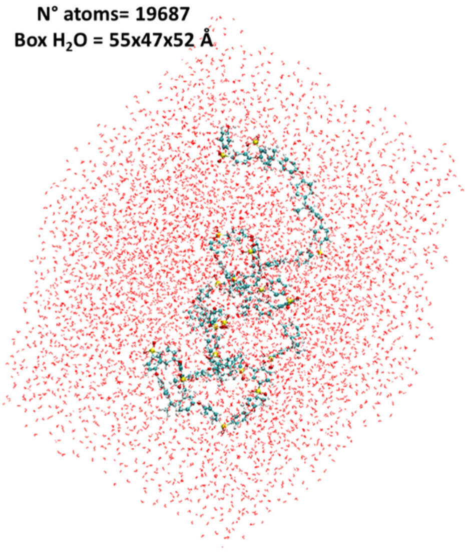

Figure S8. Preliminary single chain model, containing 10 monomers and 8 SO_3 groups.

The simulation reached the equilibrium, as highlighted by the RMSD plot reported in Figure S9. The last frame of the dynamics (Figure S9C) was adopted as initial structure for the bigger model containing 9 chains and 72 SO_3 groups, adopting the software Packmol.

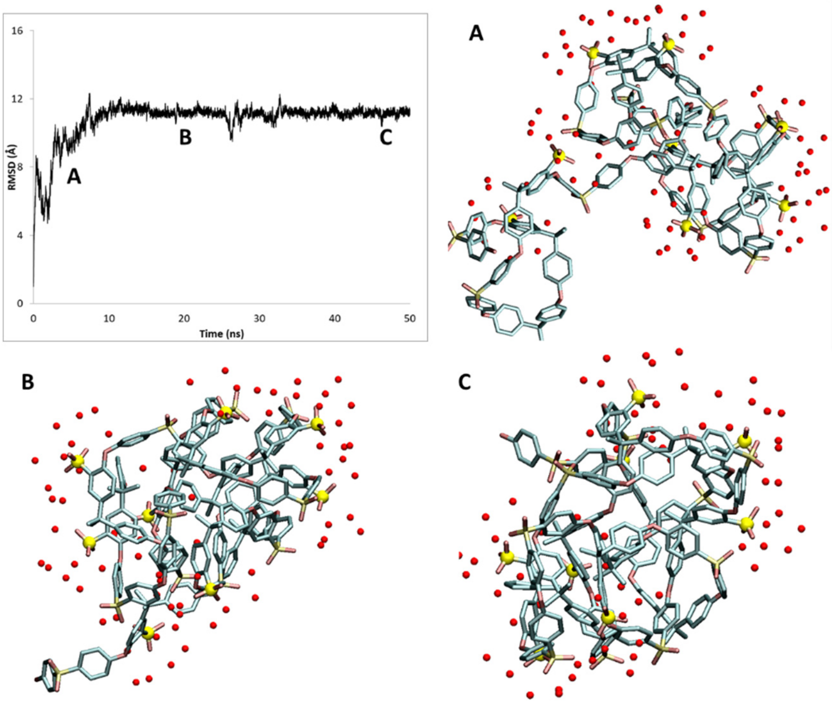

Figure S9. On the top-left, Root mean square deviation as a function of simulation time for the single chain polymer model. Selected frames (A, B, C) are also depicted. For clarity, hydrogens are not depicted. Water molecules within 5 Å from the SO_3 groups are colored in red.

In the case of the 9 chains model, the system has undergone to a longer molecular dynamics (500 ns), at the selected temperatures of 80 °C, 100 °C and 120 °C. Results about RMSD trend and representation of selected frames are reported in **Figure S10**. The frames at 500 ns have been selected to create the model including 72 monomers, described in the main manuscript.

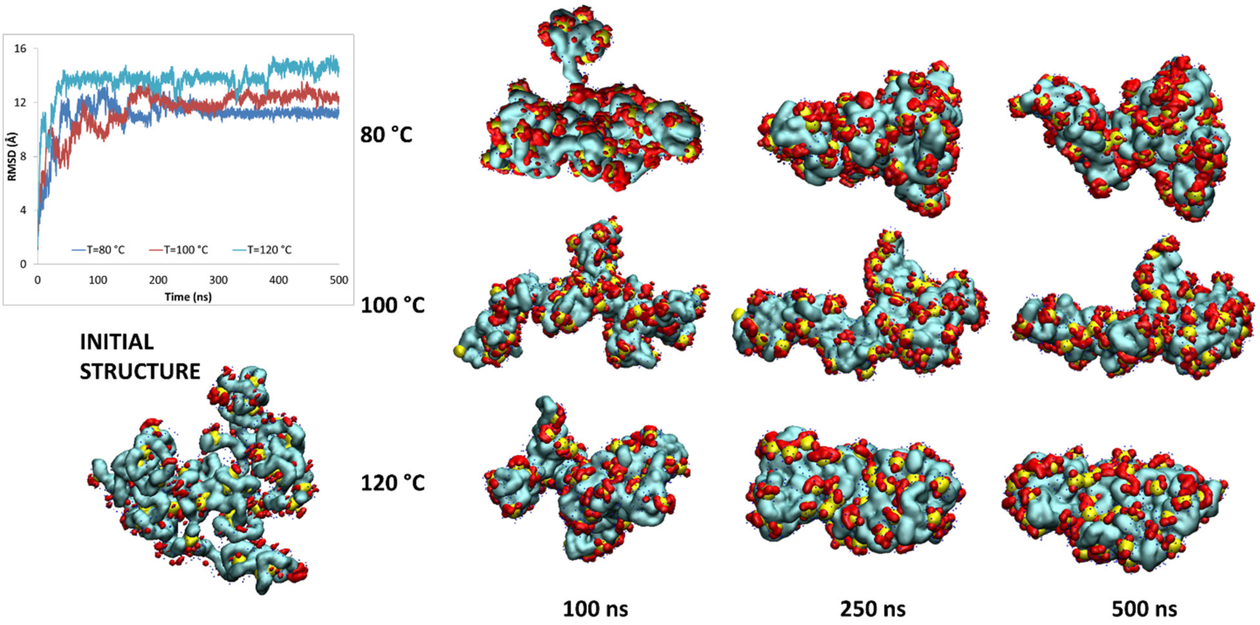

Figure S10. On the left, Root mean square deviation as a function of simulation time for the 9 chain polymer model (top) and the initial structure created starting from the single chain model (bottom). Selected frames at 100ns, 250 ns and 500 ns are also depicted. For clarity, Sulphur and atoms belonging to the polymer are colored in yellow and cyan, respectively, while water molecules within 5 Å from the SO_3 groups are represented as red surfaces.

In conclusion, the model set up can be summarized as follows:

1) Initially, MDs of single chain polymer (10 monomers, 8 SO_3 groups) has been performed to investigate behavior in solution of the molecule, at 20 °C;
2) Starting from the equilibrated structure (last frame of MDs, at 50 ns, of point 1), a model containing 9 chains (90 monomers, 72 SO_3 groups) has been created and three different MDs have been performed, at 80 °C, 100 °C and 120 °C;
3) The biggest model, consisting of 270 monomers and 216 SO_3 groups, has been finally built up starting from last frames (500 ns) and adopted for extensive MDs study presented in the manuscript.

Bibliography:

(1) Nicotera, I.; Simari, C.; Coppola, L.; Zygouri, P.; Gournis, D.; Brutti, S.; Minuto, F. D.;

Aricò, A. S.; Sebastian, D.; Baglio, V. Sulfonated Graphene Oxide Platelets in Nafion Nanocomposite Membrane: Advantages for Application in Direct Methanol Fuel Cells. *J. Phys. Chem. C* **2014**, *118* (42), 24357–24368. https://doi.org/10.1021/jp5080779.

Parameters in AMBER Force Field format

MOL.frcmod

```
remark goes here
MASS
ca 12.010        0.360
ha 1.008         0.135
os 16.000        0.465
c3 12.010        0.878
h1 1.008         0.135
hc 1.008         0.135
s6 32.060        2.900
o  16.000        0.434
sy 32.060        2.900
oh 16.000        0.465
ho 1.008         0.135

BOND
ca-ca  461.10   1.398
ca-ha  345.80   1.086
ca-os  376.60   1.370
os-c3  308.60   1.432
c3-h1  330.60   1.097
ca-c3  321.00   1.516
c3-c3  300.90   1.538
c3-hc  330.60   1.097
ca-s6  258.70   1.767
s6-o   512.70   1.453
ca-sy  243.40   1.791
sy-o   493.00   1.466
ca-oh  384.00   1.364
oh-ho  371.40   0.973

ANGLE
ca-ca-ca   66.620     120.020
ca-ca-os   69.580     119.200
ca-ca-ha   48.180     119.880
ca-os-c3   62.520     117.960
ca-ca-c3   63.530     120.770
os-c3-h1   50.800     109.780
h1-c3-h1   39.240     108.460
ca-c3-c3   63.150     112.070
ca-c3-ca   63.560     112.240
c3-c3-hc   46.340     109.800
c3-c3-c3   62.860     111.510
hc-c3-hc   39.400     107.580
ca-ca-s6   61.880     120.430
```

ca-s6-o 67.930 104.090
o -s6-o 73.590 120.050
ca-os-ca 63.480 119.890
ca-ca-sy 61.480 119.420
ca-sy-o 65.770 108.350
ca-sy-ca 60.300 104.440
o -sy-o 72.540 121.410
ca-ca-oh 69.520 119.900
ca-oh-ho 49.000 108.580

DIHE
ca-ca-ca-ca 1 3.625 180.000 2.000
ca-ca-ca-ha 1 3.625 180.000 2.000
ca-ca-os-c3 1 0.900 180.000 2.000
ca-ca-ca-c3 1 3.625 180.000 2.000
ca-os-c3-h1 1 0.383 0.000 3.000
ca-ca-ca-os 1 3.625 180.000 2.000
ca-ca-c3-c3 1 0.000 0.000 2.000
ca-ca-c3-ca 1 0.000 0.000 2.000
ha-ca-ca-ha 1 3.625 180.000 2.000
ha-ca-ca-c3 1 3.625 180.000 2.000
ha-ca-ca-os 1 3.625 180.000 2.000
ca-c3-c3-hc 1 0.156 0.000 3.000
c3-c3-c3-hc 1 0.160 0.000 3.000
ca-ca-ca-s6 1 3.625 180.000 2.000
ca-ca-s6-o 1 1.300 180.000 2.000
ca-ca-os-ca 1 0.900 180.000 2.000
s6-ca-ca-ha 1 3.625 180.000 2.000
s6-ca-ca-os 1 3.625 180.000 2.000
ca-ca-ca-sy 1 3.625 180.000 2.000
ca-ca-sy-o 1 1.300 180.000 2.000
ca-ca-sy-ca 1 1.300 180.000 2.000
ha-ca-ca-sy 1 3.625 180.000 2.000
ca-ca-ca-oh 1 3.625 180.000 2.000
ca-ca-oh-ho 1 0.900 180.000 2.000
ha-ca-ca-oh 1 3.625 180.000 2.000

IMPROPER
ca-ca-ca-ha 1.1 180.0 2.0 General improper torsional angle (2 general atom types)
ca-ca-ca-os 1.1 180.0 2.0 Using default value
c3-ca-ca-ca 1.1 180.0 2.0
ca-ca-ca-s6 1.1 180.0 2.0 Using default value
ca-ca-ca-sy 1.1 180.0 2.0 Using default value
ca-ca-ca-oh 1.1 180.0 2.0 Using default value

NONBON
ca 1.9080 0.0860
ha 1.4590 0.0150
os 1.6837 0.1700
c3 1.9080 0.1094
h1 1.3870 0.0157
hc 1.4870 0.0157
s6 2.0000 0.2500
o 1.6612 0.2100
sy 2.0000 0.2500
oh 1.7210 0.2104
ho 0.0000 0.0000

MOL.prepc

0 0 2

This is a remark line
molecule.res

```
MOL   XYZ  0
CHANGE     OMIT DU   BEG
  0.0000
   1  DUMM  DU    M       999.000     999.0      -999.0          .00000
   2  DUMM  DU    M       999.000    -999.0       999.0          .00000
   3  DUMM  DU    M      -999.000     999.0       999.0          .00000
   4  C1    ca    M       6.956000   0.074000  -1.103000  -0.259348
   5  C6    ca    B       8.001000  -0.394000  -0.318000   0.353628
   6  C5    ca    B       7.821000  -0.530000   1.041000  -0.259348
   7  C4    ca    S       6.601000  -0.193000   1.619000  -0.213013
   8  H3    ha    E       6.496000  -0.316000   2.680000   0.163297
   9  H4    ha    E       8.626000  -0.909000   1.644000   0.168563
  10  O5    os    S       9.192000  -0.748000  -0.889000  -0.427323
  11  C28   c3    3      10.081000   0.310000  -1.122000   0.037228
  12  H22   h1    E      10.967000  -0.116000  -1.573000   0.057602
  13  H23   h1    E       9.654000   1.045000  -1.796000   0.057602
  14  H24   h1    E      10.353000   0.804000  -0.193000   0.057602
  15  H1    ha    E       7.090000   0.158000  -2.167000   0.168563
  16  C2    ca    M       5.751000   0.406000  -0.519000  -0.213013
  17  H2    ha    E       4.948000   0.750000  -1.145000   0.163297
  18  C3    ca    M       5.544000   0.286000   0.858000   0.058095
  19  C7    c3    M       4.198000   0.729000   1.463000   0.341798
  20  C8    c3    3       4.054000   0.312000   2.941000  -0.447373
  21  H5    hc    E       3.072000   0.601000   3.299000   0.110212
  22  H6    hc    E       4.150000  -0.760000   3.074000   0.110212
  23  H7    hc    E       4.792000   0.801000   3.570000   0.110212
  24  C9    c3    3       4.160000   2.271000   1.422000  -0.447373
  25  H8    hc    E       4.191000   2.651000   0.408000   0.110212
  26  H9    hc    E       3.267000   2.657000   1.899000   0.110212
  27  H10   hc    E       5.021000   2.666000   1.952000   0.110212
  28  C10   ca    M       3.032000   0.093000   0.683000   0.045419
  29  C12   ca    B       1.982000   0.836000   0.163000  -0.170831
  30  C14   ca    S       0.909000   0.259000  -0.511000  -0.154592
  31  S1    s6    3      -0.375000   1.369000  -1.142000   1.209614
  32  O1    o     E       0.245000   2.677000  -1.082000  -0.662836
  33  O2    o     E      -0.658000   0.897000  -2.479000  -0.662836
  34  O3    o     E      -1.481000   1.212000  -0.210000  -0.662836
  35  H12   ha    E       1.964000   1.902000   0.243000   0.133380
  36  C11   ca    M       2.994000  -1.287000   0.503000  -0.123349
  37  H11   ha    E       3.797000  -1.903000   0.867000   0.130102
  38  C13   ca    M       1.941000  -1.884000  -0.157000  -0.345713
  39  H13   ha    E       1.913000  -2.948000  -0.307000   0.174399
  40  C15   ca    M       0.898000  -1.121000  -0.658000   0.314817
  41  O4    os    M      -0.072000  -1.798000  -1.352000  -0.299005
  42  C16   ca    M      -1.331000  -1.894000  -0.869000   0.311070
  43  C18   ca    B      -1.600000  -2.036000   0.482000  -0.264759
  44  C20   ca    S      -2.913000  -2.059000   0.910000  -0.059393
  45  H17   ha    E      -3.144000  -2.111000   1.957000   0.153074
  46  H15   ha    E      -0.797000  -2.060000   1.193000   0.181640
  47  C17   ca    M      -2.357000  -1.883000  -1.806000  -0.264759
  48  H14   ha    E      -2.105000  -1.763000  -2.841000   0.181640
  49  C19   ca    M      -3.662000  -1.919000  -1.377000  -0.059393
  50  H16   ha    E      -4.468000  -1.860000  -2.085000   0.153074
  51  C21   ca    M      -3.940000  -1.980000  -0.015000  -0.120439
  52  S2    sy    M      -5.583000  -1.716000   0.545000   1.007787
  53  O6    o     E      -5.651000  -2.082000   1.939000  -0.608291
  54  O7    o     E      -6.515000  -2.337000  -0.364000  -0.608291
  55  C22   ca    M      -5.803000   0.042000   0.394000  -0.163223
  56  C24   ca    B      -7.109000   0.526000   0.375000  -0.006424
  57  C26   ca    S      -7.345000   1.880000   0.287000  -0.395866
  58  H21   ha    E      -8.345000   2.272000   0.267000   0.192188
  59  H19   ha    E      -7.934000  -0.160000   0.417000   0.156113
  60  C23   ca    M      -4.735000   0.921000   0.310000  -0.006424
  61  H18   ha    E      -3.715000   0.591000   0.290000   0.156113
  62  C25   ca    M      -4.972000   2.282000   0.210000  -0.395866
  63  H20   ha    E      -4.130000   2.947000   0.122000   0.192188
```

```
 64  C27   ca    M        -6.269000    2.760000    0.201000    0.528598
 65  O8    oh    M        -6.550000    4.078000    0.102000   -0.653674
 66  H25   ho    E        -5.747000    4.573000    0.011000    0.445828


LOOP
  C3   C4
 C15  C14
 C21  C20
 C27  C26

IMPROPER
  C2   C6   C1   H1
  C1   C5   C6   O5
  C6   C4   C5   H4
  C3   C5   C4   H3
  C3   C1   C2   H2
  C7   C2   C3   C4
  C7  C12  C10  C11
 C10  C14  C12  H12
 C12  C15  C14   S1
 C10  C13  C11  H11
 C11  C15  C13  H13
 C14  C13  C15   O4
 C18  C17  C16   O4
 C16  C20  C18  H15
 C18  C21  C20  H17
 C16  C19  C17  H14
 C17  C21  C19  H16
 C20  C19  C21   S2
 C24  C23  C22   S2
 C22  C26  C24  H19
 C24  C27  C26  H21
 C22  C25  C23  H18
 C23  C27  C25  H20
 C26  C25  C27   O8

DONE
STOP
```

NS3.frcmod

```
remark goes here
MASS
ca 12.010       0.360
ha 1.008        0.135
os 16.000       0.465
c3 12.010       0.878
h1 1.008        0.135
hc 1.008        0.135
sy 32.060       2.900
o  16.000       0.434
oh 16.000       0.465
ho 1.008        0.135

BOND
ca-ca  461.10   1.398
ca-ha  345.80   1.086
ca-os  376.60   1.370
os-c3  308.60   1.432
c3-h1  330.60   1.097
ca-c3  321.00   1.516
c3-c3  300.90   1.538
c3-hc  330.60   1.097
```

ca-sy 243.40 1.791
sy-o 493.00 1.466
ca-oh 384.00 1.364
oh-ho 371.40 0.973

ANGLE
ca-ca-ca 66.620 120.020
ca-ca-os 69.580 119.200
ca-ca-ha 48.180 119.880
ca-os-c3 62.520 117.960
ca-ca-c3 63.530 120.770
os-c3-h1 50.800 109.780
h1-c3-h1 39.240 108.460
ca-c3-c3 63.150 112.070
ca-c3-ca 63.560 112.240
c3-c3-hc 46.340 109.800
c3-c3-c3 62.860 111.510
hc-c3-hc 39.400 107.580
ca-os-ca 63.480 119.890
ca-ca-sy 61.480 119.420
ca-sy-o 65.770 108.350
ca-sy-ca 60.300 104.440
o -sy-o 72.540 121.410
ca-ca-oh 69.520 119.900
ca-oh-ho 49.000 108.580

DIHE
ca-ca-ca-ca 1 3.625 180.000 2.000
ca-ca-ca-ha 1 3.625 180.000 2.000
ca-ca-os-c3 1 0.900 180.000 2.000
ca-ca-ca-c3 1 3.625 180.000 2.000
ca-os-c3-h1 1 0.383 0.000 3.000
ca-ca-ca-os 1 3.625 180.000 2.000
ca-ca-c3-c3 1 0.000 0.000 2.000
ca-ca-c3-ca 1 0.000 0.000 2.000
ha-ca-ca-ha 1 3.625 180.000 2.000
ha-ca-ca-c3 1 3.625 180.000 2.000
ha-ca-ca-os 1 3.625 180.000 2.000
ca-c3-c3-hc 1 0.156 0.000 3.000
c3-c3-c3-hc 1 0.160 0.000 3.000
ca-ca-os-ca 1 0.900 180.000 2.000
ca-ca-ca-sy 1 3.625 180.000 2.000
ca-ca-sy-o 1 1.300 180.000 2.000
ca-ca-sy-ca 1 1.300 180.000 2.000
ha-ca-ca-sy 1 3.625 180.000 2.000
ca-ca-ca-oh 1 3.625 180.000 2.000
ca-ca-oh-ho 1 0.900 180.000 2.000
ha-ca-ca-oh 1 3.625 180.000 2.000

IMPROPER
ca-ca-ca-ha 1.1 180.0 2.0 General improper torsional angle (2 general atom types)
ca-ca-ca-os 1.1 180.0 2.0 Using default value
c3-ca-ca-ca 1.1 180.0 2.0
ca-ca-ca-sy 1.1 180.0 2.0 Using default value
ca-ca-ca-oh 1.1 180.0 2.0 Using default value

NONBON
ca 1.9080 0.0860
ha 1.4590 0.0150
os 1.6837 0.1700
c3 1.9080 0.1094
h1 1.3870 0.0157
hc 1.4870 0.0157
sy 2.0000 0.2500
o 1.6612 0.2100
oh 1.7210 0.2104

```
 ho         0.0000  0.0000
```

NS3.frcmod

```
   0    0    2

This is a remark line
molecule.res
NS3   XYZ  0
CHANGE     OMIT DU   BEG
  0.0000
   1  DUMM  DU    M       999.000     999.0      -999.0          .00000
   2  DUMM  DU    M       999.000    -999.0       999.0          .00000
   3  DUMM  DU    M      -999.000     999.0       999.0          .00000
   4  C1    ca    M     -7.196000    1.929000   -0.669000   -0.271304
   5  C6    ca    B     -7.558000    1.627000    0.627000    0.369239
   6  C5    ca    B     -6.966000    0.547000    1.268000   -0.271304
   7  C4    ca    S     -6.028000   -0.217000    0.604000   -0.192252
   8  H3    ha    E     -5.569000   -1.038000    1.125000    0.153343
   9  H4    ha    E     -7.234000    0.326000    2.285000    0.178924
  10  O2    os    S     -8.461000    2.405000    1.286000   -0.419337
  11  C28   c3    3     -9.796000    1.968000    1.225000    0.062801
  12  H22   h1    E    -10.384000    2.678000    1.789000    0.053352
  13  H23   h1    E    -10.150000    1.941000    0.199000    0.053352
  14  H24   h1    E     -9.906000    0.981000    1.662000    0.053352
  15  H1    ha    E     -7.643000    2.776000   -1.156000    0.178924
  16  C2    ca    M     -6.250000    1.153000   -1.327000   -0.192252
  17  H2    ha    E     -5.992000    1.422000   -2.333000    0.153343
  18  C3    ca    M     -5.648000    0.065000   -0.710000    0.056494
  19  C7    c3    M     -4.631000   -0.851000   -1.418000    0.287112
  20  C8    c3    3     -5.326000   -2.203000   -1.683000   -0.453959
  21  H5    hc    E     -4.693000   -2.870000   -2.259000    0.119259
  22  H6    hc    E     -5.605000   -2.705000   -0.764000    0.119259
  23  H7    hc    E     -6.232000   -2.034000   -2.254000    0.119259
  24  C9    c3    3     -4.188000   -0.293000   -2.786000   -0.453959
  25  H8    hc    E     -3.748000    0.694000   -2.702000    0.119259
  26  H9    hc    E     -3.443000   -0.950000   -3.220000    0.119259
  27  H10   hc    E     -5.021000   -0.237000   -3.480000    0.119259
  28  C10   ca    M     -3.367000   -1.000000   -0.550000    0.036175
  29  C12   ca    B     -2.724000    0.144000   -0.071000   -0.153160
  30  C14   ca    S     -1.563000    0.067000    0.671000   -0.286635
  31  H26   ha    E     -1.085000    0.954000    1.044000    0.184915
  32  H12   ha    E     -3.148000    1.111000   -0.267000    0.148948
  33  C11   ca    M     -2.795000   -2.228000   -0.247000   -0.153160
  34  H11   ha    E     -3.249000   -3.140000   -0.582000    0.148948
  35  C13   ca    M     -1.628000   -2.321000    0.502000   -0.286635
  36  H13   ha    E     -1.202000   -3.279000    0.738000    0.184915
  37  C15   ca    M     -1.016000   -1.174000    0.952000    0.331315
  38  O1    os    M      0.105000   -1.260000    1.743000   -0.361524
  39  C16   ca    M      1.345000   -1.251000    1.206000    0.417521
  40  C18   ca    B      1.604000   -1.151000   -0.152000   -0.301739
  41  C20   ca    S      2.917000   -1.154000   -0.593000   -0.066080
  42  H17   ha    E      3.126000   -1.099000   -1.645000    0.164611
  43  H15   ha    E      0.802000   -1.082000   -0.860000    0.174524
  44  C17   ca    M      2.391000   -1.360000    2.119000   -0.301739
  45  H14   ha    E      2.160000   -1.451000    3.163000    0.174524
  46  C19   ca    M      3.693000   -1.363000    1.673000   -0.066080
  47  H16   ha    E      4.502000   -1.471000    2.370000    0.164611
  48  C21   ca    M      3.959000   -1.251000    0.311000   -0.185978
  49  S1    sy    M      5.631000   -1.247000   -0.264000    1.135866
  50  O3    o     E      5.610000   -1.668000   -1.639000   -0.598389
  51  O4    o     E      6.420000   -1.949000    0.711000   -0.598389
  52  C22   ca    M      6.153000    0.442000   -0.243000   -0.155479
  53  C24   ca    B      6.729000    0.977000    0.904000   -0.081887
```

54	C26	ca	S	7.133000	2.294000	0.925000	-0.306783
55	H21	ha	E	7.585000	2.725000	1.797000	0.183413
56	H19	ha	E	6.877000	0.356000	1.767000	0.165438
57	C23	ca	M	5.997000	1.225000	-1.374000	-0.081887
58	H18	ha	E	5.581000	0.800000	-2.267000	0.165438
59	C25	ca	M	6.402000	2.548000	-1.359000	-0.306783
60	H20	ha	E	6.288000	3.156000	-2.240000	0.183413
61	C27	ca	M	6.966000	3.082000	-0.209000	0.417213
62	O5	oh	M	7.377000	4.360000	-0.136000	-0.533847
63	H25	ho	E	7.239000	4.810000	-0.958000	0.382962

```
LOOP
  C3  C4
 C15  C14
 C21  C20
 C27  C26

IMPROPER
  C2  C6  C1  H1
  C1  C5  C6  O2
  C6  C4  C5  H4
  C3  C5  C4  H3
  C3  C1  C2  H2
  C7  C2  C3  C4
  C7  C12 C10 C11
 C10  C14 C12 H12
 C12  C15 C14 H26
 C10  C13 C11 H11
 C11  C15 C13 H13
 C14  C13 C15  O1
 C18  C17 C16  O1
 C16  C20 C18 H15
 C18  C21 C20 H17
 C16  C19 C17 H14
 C17  C21 C19 H16
 C20  C19 C21  S1
 C24  C23 C22  S1
 C22  C26 C24 H19
 C24  C27 C26 H21
 C22  C25 C23 H18
 C23  C27 C25 H20
 C26  C25 C27  O5

DONE
STOP
```

HEA-TAI.frcmod

```
remark goes here
MASS
ca 12.010       0.360
ha 1.008        0.135
c3 12.010       0.878
hc 1.008        0.135
s6 32.060       2.900
o  16.000       0.434
os 16.000       0.465
sy 32.060       2.900
oh 16.000       0.465
ho 1.008        0.135

BOND
ca-ca  461.10   1.398
```

ca-ha 345.80 1.086
ca-c3 321.00 1.516
c3-c3 300.90 1.538
c3-hc 330.60 1.097
ca-s6 258.70 1.767
s6-o 512.70 1.453
ca-os 376.60 1.370
ca-sy 243.40 1.791
sy-o 493.00 1.466
ca-oh 384.00 1.364
oh-ho 371.40 0.973

ANGLE
ca-ca-ca 66.620 120.020
ca-ca-ha 48.180 119.880
ca-ca-c3 63.530 120.770
ca-c3-c3 63.150 112.070
ca-c3-ca 63.560 112.240
c3-c3-hc 46.340 109.800
c3-c3-c3 62.860 111.510
hc-c3-hc 39.400 107.580
ca-ca-s6 61.880 120.430
ca-s6-o 67.930 104.090
ca-ca-os 69.580 119.200
o -s6-o 73.590 120.050
ca-os-ca 63.480 119.890
ca-ca-sy 61.480 119.420
ca-sy-o 65.770 108.350
ca-sy-ca 60.300 104.440
o -sy-o 72.540 121.410
ca-ca-oh 69.520 119.900
ca-oh-ho 49.000 108.580

DIHE
ca-ca-ca-ca 1 3.625 180.000 2.000
ca-ca-ca-ha 1 3.625 180.000 2.000
ca-ca-ca-c3 1 3.625 180.000 2.000
ca-ca-c3-c3 1 0.000 0.000 2.000
ca-ca-c3-ca 1 0.000 0.000 2.000
ha-ca-ca-ha 1 3.625 180.000 2.000
ha-ca-ca-c3 1 3.625 180.000 2.000
ca-c3-c3-hc 1 0.156 0.000 3.000
c3-c3-c3-hc 1 0.160 0.000 3.000
ca-ca-ca-s6 1 3.625 180.000 2.000
ca-ca-s6-o 1 1.300 180.000 2.000
ca-ca-ca-os 1 3.625 180.000 2.000
ca-ca-os-ca 1 0.900 180.000 2.000
s6-ca-ca-ha 1 3.625 180.000 2.000
s6-ca-ca-os 1 3.625 180.000 2.000
ha-ca-ca-os 1 3.625 180.000 2.000
ca-ca-ca-sy 1 3.625 180.000 2.000
ca-ca-sy-o 1 1.300 180.000 2.000
ca-ca-sy-ca 1 1.300 180.000 2.000
ha-ca-ca-sy 1 3.625 180.000 2.000
ca-ca-ca-oh 1 3.625 180.000 2.000
ca-ca-oh-ho 1 0.900 180.000 2.000
ha-ca-ca-oh 1 3.625 180.000 2.000

IMPROPER
ca-ca-ca-ha 1.1 180.0 2.0 General improper torsional angle (2 general atom types)
c3-ca-ca-ca 1.1 180.0 2.0
ca-ca-ca-s6 1.1 180.0 2.0 Using default value
ca-ca-ca-os 1.1 180.0 2.0 Using default value
ca-ca-ca-sy 1.1 180.0 2.0 Using default value
ca-ca-ca-oh 1.1 180.0 2.0 Using default value

```
NONBON
  ca         1.9080  0.0860
  ha         1.4590  0.0150
  c3         1.9080  0.1094
  hc         1.4870  0.0157
  s6         2.0000  0.2500
  o          1.6612  0.2100
  os         1.6837  0.1700
  sy         2.0000  0.2500
  oh         1.7210  0.2104
  ho         0.0000  0.0000
```

HEA.frcmod

```
    0    0    2

This is a remark line
molecule.res
HEA   XYZ  0
CHANGE     OMIT DU   BEG
  0.0000
   1  DUMM  DU    M       999.000     999.0     -999.0          .00000
   2  DUMM  DU    M       999.000    -999.0      999.0          .00000
   3  DUMM  DU    M      -999.000     999.0      999.0          .00000
   4  C1    ca    M      7.497000   0.060000  -1.391000  -0.145627
   5  C6    ca    B      8.554000  -0.446000  -0.647000  -0.153458
   6  C5    ca    B      8.400000  -0.600000   0.716000  -0.145627
   7  C4    ca    S      7.205000  -0.249000   1.335000  -0.213978
   8  H3    ha    E      7.123000  -0.383000   2.396000   0.147571
   9  H4    ha    E      9.207000  -0.994000   1.310000   0.133065
  10  H23   ha    E      9.477000  -0.718000  -1.127000   0.127741
  11  H1    ha    E      7.595000   0.181000  -2.456000   0.133065
  12  C2    ca    M      6.310000   0.404000  -0.774000  -0.213978
  13  H2    ha    E      5.498000   0.778000  -1.370000   0.147571
  14  C3    ca    M      6.138000   0.259000   0.605000   0.136246
  15  C7    c3    M      4.814000   0.711000   1.251000   0.265620
  16  C8    c3    3      4.708000   0.294000   2.732000  -0.469108
  17  H5    hc    E      3.741000   0.593000   3.118000   0.120900
  18  H6    hc    E      4.797000  -0.779000   2.861000   0.120900
  19  H7    hc    E      5.470000   0.775000   3.339000   0.120900
  20  C9    c3    3      4.785000   2.253000   1.211000  -0.469108
  21  H8    hc    E      4.784000   2.635000   0.197000   0.120900
  22  H9    hc    E      3.909000   2.644000   1.718000   0.120900
  23  H10   hc    E      5.664000   2.642000   1.714000   0.120900
  24  C10   ca    M      3.623000   0.082000   0.503000   0.070970
  25  C12   ca    B      2.558000   0.829000   0.021000  -0.155857
  26  C14   ca    S      1.463000   0.257000  -0.621000  -0.164785
  27  S1    s6    3      0.160000   1.372000  -1.205000   1.201833
  28  O1    o     E      0.783000   2.679000  -1.158000  -0.659742
  29  O2    o     E     -0.166000   0.907000  -2.534000  -0.659742
  30  O3    o     E     -0.915000   1.211000  -0.239000  -0.659742
  31  H12   ha    E      2.546000   1.895000   0.106000   0.128172
  32  C11   ca    M      3.575000  -1.298000   0.319000  -0.134253
  33  H11   ha    E      4.388000  -1.917000   0.655000   0.127991
  34  C13   ca    M      2.500000  -1.890000  -0.310000  -0.341525
  35  H13   ha    E      2.466000  -2.953000  -0.464000   0.174245
  36  C15   ca    M      1.444000  -1.122000  -0.774000   0.314648
  37  O4    os    M      0.451000  -1.794000  -1.440000  -0.299637
  38  C16   ca    M     -0.792000  -1.891000  -0.916000   0.311670
  39  C18   ca    B     -1.016000  -2.040000   0.442000  -0.265750
  40  C20   ca    S     -2.316000  -2.064000   0.913000  -0.059907
  41  H17   ha    E     -2.512000  -2.120000   1.966000   0.153670
```

```
 42  H15   ha    E        -0.191000   -2.068000    1.126000    0.182298
 43  C17   ca    M        -1.848000   -1.875000   -1.819000   -0.265750
 44  H14   ha    E        -1.630000   -1.750000   -2.861000    0.182298
 45  C19   ca    M        -3.138000   -1.912000   -1.349000   -0.059907
 46  H16   ha    E        -3.966000   -1.848000   -2.030000    0.153670
 47  C21   ca    M        -3.371000   -1.979000    0.022000   -0.122257
 48  S2    sy    M        -4.995000   -1.715000    0.636000    1.015395
 49  O5    o     E        -5.019000   -2.088000    2.030000   -0.610412
 50  O6    o     E        -5.957000   -2.330000   -0.246000   -0.610412
 51  C22   ca    M        -5.217000    0.044000    0.501000   -0.167446
 52  C24   ca    B        -6.521000    0.531000    0.528000   -0.006202
 53  C26   ca    S        -6.758000    1.885000    0.453000   -0.395004
 54  H21   ha    E        -7.757000    2.278000    0.469000    0.191855
 55  H19   ha    E        -7.346000   -0.155000    0.594000    0.156574
 56  C23   ca    M        -4.151000    0.922000    0.386000   -0.006202
 57  H18   ha    E        -3.132000    0.591000    0.330000    0.156574
 58  C25   ca    M        -4.388000    2.284000    0.300000   -0.395004
 59  H20   ha    E        -3.548000    2.948000    0.188000    0.191855
 60  C27   ca    M        -5.684000    2.764000    0.337000    0.528240
 61  O7    oh    M        -5.966000    4.082000    0.254000   -0.653517
 62  H22   ho    E        -5.166000    4.577000    0.139000    0.445701


LOOP
  C3   C4
 C15  C14
 C21  C20
 C27  C26

IMPROPER
  C2   C6   C1   H1
  C1   C5   C6  H23
  C6   C4   C5   H4
  C3   C5   C4   H3
  C3   C1   C2   H2
  C7   C2   C3   C4
  C7  C12  C10  C11
 C10  C14  C12  H12
 C12  C15  C14   S1
 C10  C13  C11  H11
 C11  C15  C13  H13
 C14  C13  C15   O4
 C18  C17  C16   O4
 C16  C20  C18  H15
 C18  C21  C20  H17
 C16  C19  C17  H14
 C17  C21  C19  H16
 C20  C19  C21   S2
 C24  C23  C22   S2
 C22  C26  C24  H19
 C24  C27  C26  H21
 C22  C25  C23  H18
 C23  C27  C25  H20
 C26  C25  C27   O7

DONE
STOP
```

TAI.frcmod

```
   0    0    2

This is a remark line
molecule.res
```

```
TAI XYZ 0
CHANGE OMIT DU BEG
 0.0000
 1 DUMM DU M 999.000 999.0 -999.0 .00000
 2 DUMM DU M 999.000 -999.0 999.0 .00000
 3 DUMM DU M -999.000 999.0 999.0 .00000
 4 C1 ca M 7.497000 0.060000 -1.391000 -0.145627
 5 C6 ca B 8.554000 -0.446000 -0.647000 -0.153458
 6 C5 ca B 8.400000 -0.600000 0.716000 -0.145627
 7 C4 ca S 7.205000 -0.249000 1.335000 -0.213978
 8 H3 ha E 7.123000 -0.383000 2.396000 0.147571
 9 H4 ha E 9.207000 -0.994000 1.310000 0.133065
 10 H23 ha E 9.477000 -0.718000 -1.127000 0.127741
 11 H1 ha E 7.595000 0.181000 -2.456000 0.133065
 12 C2 ca M 6.310000 0.404000 -0.774000 -0.213978
 13 H2 ha E 5.498000 0.778000 -1.370000 0.147571
 14 C3 ca M 6.138000 0.259000 0.605000 0.136246
 15 C7 c3 M 4.814000 0.711000 1.251000 0.265620
 16 C8 c3 3 4.708000 0.294000 2.732000 -0.469108
 17 H5 hc E 3.741000 0.593000 3.118000 0.120900
 18 H6 hc E 4.797000 -0.779000 2.861000 0.120900
 19 H7 hc E 5.470000 0.775000 3.339000 0.120900
 20 C9 c3 3 4.785000 2.253000 1.211000 -0.469108
 21 H8 hc E 4.784000 2.635000 0.197000 0.120900
 22 H9 hc E 3.909000 2.644000 1.718000 0.120900
 23 H10 hc E 5.664000 2.642000 1.714000 0.120900
 24 C10 ca M 3.623000 0.082000 0.503000 0.070970
 25 C12 ca B 2.558000 0.829000 0.021000 -0.155857
 26 C14 ca S 1.463000 0.257000 -0.621000 -0.164785
 27 S1 s6 3 0.160000 1.372000 -1.205000 1.201833
 28 O1 o E 0.783000 2.679000 -1.158000 -0.659742
 29 O2 o E -0.166000 0.907000 -2.534000 -0.659742
 30 O3 o E -0.915000 1.211000 -0.239000 -0.659742
 31 H12 ha E 2.546000 1.895000 0.106000 0.128172
 32 C11 ca M 3.575000 -1.298000 0.319000 -0.134253
 33 H11 ha E 4.388000 -1.917000 0.655000 0.127991
 34 C13 ca M 2.500000 -1.890000 -0.310000 -0.341525
 35 H13 ha E 2.466000 -2.953000 -0.464000 0.174245
 36 C15 ca M 1.444000 -1.122000 -0.774000 0.314648
 37 O4 os M 0.451000 -1.794000 -1.440000 -0.299637
 38 C16 ca M -0.792000 -1.891000 -0.916000 0.311670
 39 C18 ca B -1.016000 -2.040000 0.442000 -0.265750
 40 C20 ca S -2.316000 -2.064000 0.913000 -0.059907
 41 H17 ha E -2.512000 -2.120000 1.966000 0.153670
 42 H15 ha E -0.191000 -2.068000 1.126000 0.182298
 43 C17 ca M -1.848000 -1.875000 -1.819000 -0.265750
 44 H14 ha E -1.630000 -1.750000 -2.861000 0.182298
 45 C19 ca M -3.138000 -1.912000 -1.349000 -0.059907
 46 H16 ha E -3.966000 -1.848000 -2.030000 0.153670
 47 C21 ca M -3.371000 -1.979000 0.022000 -0.122257
 48 S2 sy M -4.995000 -1.715000 0.636000 1.015395
 49 O5 o E -5.019000 -2.088000 2.030000 -0.610412
 50 O6 o E -5.957000 -2.330000 -0.246000 -0.610412
 51 C22 ca M -5.217000 0.044000 0.501000 -0.167446
 52 C24 ca B -6.521000 0.531000 0.528000 -0.006202
 53 C26 ca S -6.758000 1.885000 0.453000 -0.395004
 54 H21 ha E -7.757000 2.278000 0.469000 0.191855
 55 H19 ha E -7.346000 -0.155000 0.594000 0.156574
 56 C23 ca M -4.151000 0.922000 0.386000 -0.006202
 57 H18 ha E -3.132000 0.591000 0.330000 0.156574
 58 C25 ca M -4.388000 2.284000 0.300000 -0.395004
 59 H20 ha E -3.548000 2.948000 0.188000 0.191855
 60 C27 ca M -5.684000 2.764000 0.337000 0.528240
 61 O7 oh M -5.966000 4.082000 0.254000 -0.653517
 62 H22 ho E -5.166000 4.577000 0.139000 0.445701
```

```
LOOP
  C3   C4
 C15  C14
 C21  C20
 C27  C26

IMPROPER
  C2   C6   C1   H1
  C1   C5   C6  H23
  C6   C4   C5   H4
  C3   C5   C4   H3
  C3   C1   C2   H2
  C7   C2   C3   C4
  C7  C12  C10  C11
 C10  C14  C12  H12
 C12  C15  C14   S1
 C10  C13  C11  H11
 C11  C15  C13  H13
 C14  C13  C15   O4
 C18  C17  C16   O4
 C16  C20  C18  H15
 C18  C21  C20  H17
 C16  C19  C17  H14
 C17  C21  C19  H16
 C20  C19  C21   S2
 C24  C23  C22   S2
 C22  C26  C24  H19
 C24  C27  C26  H21
 C22  C25  C23  H18
 C23  C27  C25  H20
 C26  C25  C27   O7

DONE
STOP
```
